# Editorial: The primate's sensorimotor system and its relationship with emotion, cognition, and decision-making

**DOI:** 10.3389/fnint.2025.1629851

**Published:** 2025-06-05

**Authors:** Luciano Simone, Marzio Gerbella, Luca Fornia

**Affiliations:** ^1^Department of Medicine and Surgery, University of Parma, Parma, Italy; ^2^Department of Medical Biotechnology and Translational Medicine, University of Milan, Milan, Italy; ^3^IRCCS Fondazione Don Carlo Gnocchi ONLUS, Milan, Italy

**Keywords:** integrative neuroscience, sensorimotor control, comparative cognition, parietofrontal circuits, comparative behavior

Primates' capacity to adapt to complex environments arises from the advanced integration of sensorimotor, cognitive, and affective systems. Rather than operating in isolation, the sensorimotor system is now recognized as dynamically interconnected with brain regions involved in higher-order cognitive functions and emotional regulation.

Contemporary neuroscience increasingly reveals a sophisticated, cross-domain network in which action is continuously shaped by cognitive and affective processes. The six studies in this Research Topic, “*The primate's sensorimotor system and its relationship with emotion, cognition, and decision-making,”* underscore the importance of understanding the link between cognition and sensorimotor function to trace the evolutionary trajectory from non-human primates to humans.

In line with this framework, results from Ishida et al., based on neuronal recordings in monkeys, reveal that regions within the posterior perisylvian sulcus, the secondary somatosensory cortex, and the posterior insular cortex, once thought to be exclusively involved in passive sensory integration, actually play a critical role in sensorimotor integration during grasping and object manipulation. The authors suggest that these findings support the hypothesis that these areas are involved in complex sensorimotor processes subserving predictive modeling and attentional modulation.

These discoveries align with the longstanding view, supported by extensive neuroimaging and electrophysiological evidence, that the insula has a complex functional profile and participates in several sensorimotor networks. Accordingly, a study within this Research Topic by Sypré et al. employed a data-driven clustering approach to subdivide the macaque insula into fields based on the similarity of each voxel's functional connectivity profile. Specifically, this approach enabled the attribution of each insular field to relatively distinct functional circuits: the anterior field was linked to mouth motor and gustatory networks; the intermediate dorsal field to the grasping network; the intermediate ventral field to regions involved in social and affiliative processing; and the posterior field to optic flow and vestibular networks. By integrating these resting-state-derived subdivisions with task-based and seed-to-brain fMRI results, the authors also demonstrated specialized roles of the various insular fields in gustatory, somatomotor, vestibular, and social visual processing.

In addition to the insular cortex, another brain region recently shown to be involved in many sensorimotor processes, and whose expansion in both humans and non-human primates has significantly contributed to the sophistication of the sensorimotor repertoire, is the lateral prefrontal cortex (LPF). Neuroanatomical studies have revealed that the caudal regions of the LPF connect mainly to parietal and prearcuate oculomotor areas, the middle regions to parietal and frontal skeletomotor areas, and the rostral region chiefly to other prefrontal areas. To explore whether this LPF connectional organization is reflected in subcortical structures, Borra et al. examined the projection patterns from the mediodorsal thalamic nucleus (MD) to the LPF. Coherently with the hypothesis, the study revealed a topographic organization of LPF projections to the MD that mirrors the aforementioned rostrocaudal gradient.

These results highlight how distributed thalamocortical circuits support outcome monitoring, rule implementation, and sensorimotor transformation, delineating specific subcortical routes through which cognition exerts top-down modulation over motor behavior.

While the prefrontal cortex is an associative region where abstract rules are used to guide appropriate context dependent behaviors, the posterior parietal cortex, whose late evolutionary expansion parallels the increasing sensorimotor complexity of primate behavior, serves as a central hub for integration sensory and action information. Within this cortex, the lateral intraparietal area (LIP) is part of a circuit involved in the transformation of visual inputs into coordinated saccadic eye movements. Brunamonti and Paré recorded LIP neuronal activity in rhesus monkeys performing both a delayed saccade task and a countermanding task, requiring either execution or inhibition of a planned saccade after a stop signal, to further clarify the role of LIP in oculomotor control. They found that LIP activity did not modulate during the critical window for saccade cancellation, suggesting that LIP serves primarily as a hub for attentional mapping and corollary discharge, rather than as a direct motor initiator.

Together, these studies demonstrate that the sensorimotor system operates within a distributed, hierarchically organized network that enables real-time interaction between physical action, emotional salience, and cognitive load, thus redefining what it means for a brain region to be “motor” or “sensory.”

The goal of studies on the monkey brain is to translate the knowledge gained into understanding the human species, as non-human primates represent our closest evolutionary relatives. They possess remarkable manual abilities and live in complex social environments, making them highly valuable models for investigating the neural mechanisms underlying action, cognition, and social interaction. In this respect, Gambaretti et al. and Tariciotti et al. provide critical syntheses that extend the experimental findings on monkeys into clinical domains. The first study underscores how foundational research in non-human primates has shaped the development of intraoperative brain mapping, particularly through refined electrical stimulation techniques aimed at preserving motor functions during tumor resection. The second offers a systematic synthesis of the neural substrates underlying object-oriented hand dexterity across species, bridging functional imaging with recent data obtained in awake neurosurgical patients. Together, they outline how the distributed nature of sensorimotor control, grounded in comparative primate studies, has transformed neurosurgical approaches, particularly in the preservation of corticospinal pathways and parietofrontal circuits subserving praxis, executive functions, and goal-directed motor abilities during glioma resection.

Localizing and preserving these anatomo-functional building blocks during neurosurgery is crucial for ensuring post-operative quality of life. Their work underscores a key message: evolutionarily conserved structures have been co-opted and elaborated in humans to subserve higher-order cognition and complex motor behaviors.

In summary, the six studies featured in this Research Topic collectively emphasize that the sensorimotor system is not merely a means of interacting with the external world, but a dynamic interface where action and multiple cognitive domains converge. On one hand, the presence of such integrative, large-scale neural architectures in both human and non-human primates reinforces the principle of evolutionary continuity; on the other, the expansion and reorganization of these neural networks in humans underpin the emergence of uniquely human socio-cognitive behaviors. Understanding this duality, of conservation and divergence, is essential to understanding ourselves.

